# Identification of women with an increased risk of developing radiation-induced breast cancer: a case only study

**DOI:** 10.1186/bcr1668

**Published:** 2007-04-11

**Authors:** Annegien Broeks, Linde M Braaf, Angelina Huseinovic, Anke Nooijen, Jos Urbanus, Frans BL Hogervorst, Marjanka K Schmidt, Jan GM Klijn, Nicola S Russell, Flora E Van Leeuwen, Laura J Van 't Veer

**Affiliations:** 1 Division of Experimental Therapy, The Netherlands Cancer Institute, Plesmanlaan 121, 1066 CX Amsterdam, The Netherlands; 2 Department of Pathology, The Netherlands Cancer Institute, Plesmanlaan 121, 1066 CX Amsterdam, The Netherlands; 3 Department of Epidemiology, The Netherlands Cancer Institute, Plesmanlaan 121, 1066 CX Amsterdam, The Netherlands; 4 Department of Medical Oncology, Dr Daniel den Hoed Cancer Centre, Groenehilledijk 301, 3075 EA, Rotterdam, The Netherlands; 5 Department of Radiotherapy, The Netherlands Cancer Institute, Plesmanlaan 121, 1066 CX Amsterdam, The Netherlands

## Abstract

**Introduction:**

Radiation exposure at a young age is one of the strongest risk factors for breast cancer. Germline mutations in genes involved in the DNA-damage repair pathway (DDRP) may render women more susceptible to radiation-induced breast cancer.

**Methods:**

We evaluated the contribution of germline mutations in the DDRP genes *BRCA1*, *BRCA2*, *CHEK2 *and *ATM *to the risk of radiation-induced contralateral breast cancer (CBC). The germline mutation frequency was assessed, in a case-only study, in women who developed a CBC after they had a first breast cancer diagnosed before the age of 50 years, and who were (*n *= 169) or were not (*n *= 78) treated with radiotherapy for their first breast tumour.

**Results:**

We identified 27 *BRCA1*, 5 *BRCA2*, 15 *CHEK2 *and 4 truncating *ATM *germline mutation carriers among all CBC patients tested (21%). The mutation frequency was 24.3% among CBC patients with a history of radiotherapy, and 12.8% among patients not irradiated for the first breast tumour (odds ratio 2.18 (95% confidence interval 1.03 to 4.62); *p *= 0.043). The association between DDRP germline mutation carriers and risk of radiation-induced CBC seemed to be strongest in women who developed their second primary breast tumour at least 5 years after radiotherapy. Those patients had an odds ratio of 2.51 (95% confidence interval 1.03 to 6.10; *p *= 0.049) of developing radiation-induced breast cancer, in comparison with non-carriers.

**Conclusion:**

This study shows that carriers of germline mutations in a DDRP gene have an increased risk of developing (contralateral) breast cancer after radiotherapy; that is, over and above the risk associated with their carrier status. The increased risk indicates that knowledge of germline status of these DDRP genes at the time of breast cancer diagnosis may have important implications for the choice of treatment.

## Introduction

Several risk factors for the development of breast cancer, such as family history, reproductive factors and exposure to radiation, have been identified. Out of all breast cancers, 5 to 10% can be attributed to germline mutations in familial high-risk genes such as *BRCA1 *or *BRCA2 *that result in a lifetime breast cancer risk of about 45 to 65% [1]. The penetrance varies between families, depending on 'risk modifiers' such as hormonal factors, mutation type and also exposure to radiation [2-4]. Mutations in low-penetrance genes may account for a larger proportion (10 to 30%) of all breast cancers [5]. The contribution of these genes might be explained by their role in the DNA-damage control pathway. For instance, *ATM *heterozygous carriers have a relative risk of breast cancer of 2.2 compared with the general population and a relative risk of 4.9 for those younger than 50 years of age [6,7]. It has been shown that particular alterations in the *ATM *gene are associated with increased *in vitro *chromosomal sensitivity to radiation [8-10]. One particular sequence variant in CHEK2 (CHEK2*1100delC) has been implicated in a twofold increased risk of breast cancer, functioning as a low-penetrance breast cancer susceptibility allele [11-15]. Mutation frequencies reported in the literature indicate that about 10% of all women with breast cancer diagnosed before the age of 50 years have a germline mutation in *BRCA1*, *BRCA2*, *ATM *or *CHEK2 *[1,6,15].

Exposure to ionising radiation is a strong risk factor for breast cancer. A pooled analysis of eight cohorts by Preston and colleagues showed that the increased risk is directly proportional to the radiation dose received and inversely related to age at irradiation [16]. For women exposed at the age of 25 years the excess relative risk per Gy was estimated to be 1.8. Radiation-induced DNA damage initiates a complex series of overlapping responses responsible for the maintenance of genome integrity. The increased incidence of breast cancer after exposure to ionising radiation might be restricted to a genetically defined radiosensitive subpopulation [17]. Candidate genes (for example *BRCA1*, *BRCA2*, *CHEK2*, *ATM*, *MDM2 *and *TP53*) are implicated in the maintenance of genome integrity; their involvement in breast cancer susceptibility and their role in DNA-damage repair signalling make them excellent candidates as genes with a role in radiation-induced breast cancer [18].

The use of diagnostic X-rays has not been associated with increased risk of breast cancer in the general population, with the exception of frequent chest fluoroscopies in tuberculosis [19,20]. However, low-dose ionising radiation has recently been shown to increase the risk of breast cancer significantly among *BRCA1 *and *BRCA2 *mutation carriers [4]. In a retrospective cohort study of 1,601 female *BRCA1 *and *BRCA2 *mutation carriers we found an association with reported chest X-ray exposure and significantly increased risk of breast cancer (hazard ratio 1.54). Furthermore, a recent publication also showed a strong association (odds ratio (OR) 3.21) between CHEK2*1100delC carrier status, breast cancer risk and a history of chest X-rays [21].

Women with breast cancer in general have a threefold to fourfold increased risk of developing a new primary cancer in the opposite breast [22]. The increased risk may be explained by the same genetic and hormonal factors that caused the first breast cancer. In addition, radiotherapy for primary breast cancer may also contribute to the development of cancer in the contralateral breast. The contralateral breast can receive a dose of several Gy of leakage and scattered radiation during radiation treatment (RT) [23,24]. Several studies have shown that exposure of the contralateral breast to RT increases the risk of developing second primary breast cancer among young women [23,25].

To evaluate the association between germline mutations in DNA-damage repair pathway (DDRP) genes (*BRCA1*, *BRCA2*, *CHEK2 *and *ATM*) and radiation-induced contralateral breast cancer (CBC), we conducted a case-only study. We assessed the germline mutation frequency in women who developed CBC, according to their history of RT for the first breast cancer, and assessed whether DDRP mutation carriers have an increased risk of radiation-associated breast cancer compared with that for non-carriers.

## Methods

### Patients

We examined the interaction of germline mutation status and exposure to radiotherapy in the pathogenesis of CBC in a case-only design. Such an approach is especially suitable for the evaluation of gene-environment interactions [26,27]. The consecutive breast cancer patients included in this study were all selected from the hospital tumour registries of The Netherlands Cancer Institute (NKI-AVL), Amsterdam, and the Dr Daniel den Hoed Cancer Centre/Erasmus Medical Center (DDHK), Rotterdam. We achieved an 80% response rate from all patients who were invited to participate. CBC patients (with a histologically confirmed second primary breast tumour) were included if their first breast cancer was diagnosed before the age of 50 years (between 1966 and 2000) and the second breast cancer was diagnosed at least 1 year later (*n *= 247).

One hundred and sixty-nine patients had received radiotherapy for their primary invasive breast tumour and 78 had not (main indications for radiotherapy were lymph-node status and prognosis at the time of diagnosis). All patients had been treated with surgery, 23% had had chemotherapy in addition to RT, and 9% had received chemotherapy in the no-RT group (see also Table 1). Patients who were treated with radiotherapy received one or more radiation fields with either kilovolt or megavolt radiation quality. The dose to the ipsilateral primary tumour site varied from 30.5 Gy (internal mammary chain irradiation) to 76 Gy (breast-conserving treatment with a boost dose). The contralateral breast received about 1 to 10% from scatter and collimator leakage (this problem continues despite modern radiation methods). The maximum radiation dose at the contralateral breast and the dose at the site of the contralateral tumour were estimated from the treatment charts by a radiation oncologist (NS Russell; details of the calculation of the radiation dose to the contralateral breast (taking into account all given doses, fields, machine types, scatter and collimator leakage) are available from the authors on request).

Detailed treatment data, disease characteristics and patient characteristics were obtained from medical records and risk factor questionnaires (Table 1). Patients were asked to donate a 20-ml blood sample or permission for the use of paraffin-embedded tissue blocks, and all patients gave written informed consent for mutation analysis. This study received approval from the Medical Ethical Committees of NKI-AVL and DDHK.

### Genomic DNA isolation

Genomic DNA was either isolated from peripheral blood lymphocytes with the use of DNAzol (Invitrogen, Breda, The Netherlands) methods in accordance with the manufacturer's instructions, or from three 10-μm paraffin normal tissue slides in accordance with standard protocols [28]. For histopathological examination of the tumour we used a haematoxylin/eosin-stained slide.

### Mutation analysis

The complete *ATM *open reading frame was analysed; each exon (exons 4 to 65) and corresponding splice sites were screened for germline mutations by denaturing gradient gel electrophoresis, identifying 80 to 90% of all *ATM *mutations and polymorphisms [29]. Detection of the CHEK2*1100delC mutation was performed by using denaturing gradient gel electrophoresis. All primers were designed with the Ingeny melt analysis software; and primer sequences are available from the authors on request. The products were analysed on a polyacrylamide/20 to 55% urea/formamide gradient gel and run overnight at 120 V and 59°C.

Using DSDI (detection of small deletions and insertions; genotype analysis on an ABI Prism 3700 DNA analyzer and corresponding software) and allelic discrimination (AD; with Taqman probes using a ABI Prism 7700 sequence detector) we screened for *BRCA1 *and *BRCA2 *mutations. We chose the fragments for DSDI and the ADs as those in which we were able to detect the most frequently occurring known pathogenic mutations, including Dutch founder mutations, on the basis of data from *BRCA1/2 *screening in breast cancer families and in young breast cancer patients from almost all clinical genetic centres in The Netherlands. In total we screened for 32 different mutations in *BRCA1 *and 16 in *BRCA2*, which represented at the time of analysis about 81% and about 39% of known mutations occurring in Dutch breast cancer families. Methods are available from the authors on request.

All aberrations were confirmed by genomic sequence analysis. Sequence analysis was performed with the ABI PRISM Big-DyeTerminator Cycle Sequencing Ready Reaction Kit, Version 3.1 (Applied Biosystems, Nieuwerkerk a/d Ijssel, The Netherlands). Sequencing products were analysed with the ABI Prism 3700 DNA analyser and corresponding software.

### Statistical analysis

Statistical analyses were performed by using standard methods for the analysis of case-control studies [30]. We compared the mutation frequency between CBC cases previously treated with radiation and those not treated with radiation. ORs and 95% confidence intervals (CIs) were calculated to evaluate the association between radiation exposure, mutation carrier status and breast cancer risk. Logistic regression was performed to examine the effect of potential confounders (age at first breast cancer, tumour size, lymph node status and family history) on risk estimates. Under the assumption that there is independence between genotype and RT, the OR estimates the relative risk of CBC associated with radiation among gene carriers compared with the relative risk of CBC associated with radiation among non-carriers. All analyses were performed with SPSS 12.0 (SPSS Inc., Chicago, IL, USA).

## Results

### Pathogenic and missense mutation frequencies

For all CBC patients we obtained germline mutation data for the DDRP genes *BRCA1*, *BRCA2*, *CHEK2 *and *ATM*. In total, we identified 51 pathogenic germline mutations in these DDRP genes among the 247 CBC patients (21%), an increase over the estimated 10% mutation carriers among all women with primary breast cancer diagnosed before the age of 50 years (estimated from mutation frequencies reported previously [1,6,15]; Table 2). We did not detect multiple pathogenic mutations in single patients. We compared the mutation frequency between CBC cases previously treated with radiation and those not treated with radiation. Among women who had received radiotherapy for their first breast cancer (*n *= 169), we identified 24.3% mutation carriers, in contrast with only 12.8% among those who had not received radiotherapy (*n *= 78). Our data show that women with a pathogenic mutation in one of the tested genes have a 2.18-fold increased risk (95% CI 1.03 to 4.62) of developing CBC after radiotherapy for the first breast cancer, compared with women without a pathogenic mutation. The pattern of a higher proportion of carriers among the CBC cases with previous RT than among those with no previous RT was also found for each of the individual genes, although these results did not reach significance.

The mean age at diagnosis of the first primary breast cancer was 41 years in the CBC group with previous RT and 42 years for CBC patients not treated with radiation (Table 1). Furthermore, the mean age at diagnosis of the first primary breast cancer was 41 years in the mutation carrier group (interquartile range 36 to 47 years) and 42 years in the non-carriers (interquartile range 38 to 47 years), resulting in the same mean (and median) age at time of treatment in both groups. Adjustment for age in logistic regression analysis did not materially affect the risk estimates: the adjusted OR was 2.14 (95% CI 1.01 to 4.55). DDRP mutation carriers had clinico-pathological features that were similar to those of 'sporadic' cases. Tumour size, lymph node status and type of tumour were comparable in the mutation-positive and mutation-negative groups (Table 1). Adjustment for each of these three factors in logistic regression analysis did not materially affect the risk estimates for mutation status.

In addition to the proven *ATM *pathogenic truncating mutations we also detected in *ATM *a large number of presumed neutral polymorphisms and missense mutations (Table 3). Forty-three individuals carried at least one *ATM *missense mutation, of whom 7 had multiple *ATM *missense mutations [31]. Moreover, 14 patients had a pathogenic mutation in one of the tested DDRP genes in addition to an *ATM *missense mutation. Overall, we did not detect a significantly increased proportion of women with missense mutations among those who had received RT compared with those who had not (OR 1.24 (95% CI 0.52 to 2.94)). Although these data are also not statistically significant, there might be an increased risk of developing radiation-induced breast cancer in those women carrying a combination of both a pathogenic DDRP and an *ATM *missense mutation (OR 2.90 (95% CI 0.63 to 13.30); Table 3).

The literature [6,41] shows that *ATM *missense mutation carriers have an increased risk for breast cancer. Because there is no conclusive evidence for the function of any of these missense mutations yet, we did not take these missense mutations into account in the subsequent analysis exploring the effect of RT.

### Effects of age at radiation, radiation dose, and induction period

Because age at radiation is a major determinant of breast cancer risk, we examined whether younger age at irradiation was associated with a greater radiation effect in carriers. All women in our study had their first breast tumour diagnosed before the age of 50 years, but none of them had cancer before they were 20 years old. We divided the cases by age at radiation into those exposed below the age of 40 years and those exposed between 40 and 50 years of age. Although the results were not significant, they showed that the radiation effect might be more pronounced in the younger mutation carriers at exposure (Table 4).

The contralateral breast received about 1 to 10% of the total dose from scatter and collimator leakage from the RT machine. To investigate whether germline pathological mutations most strongly affect CBC risk in women who had highest levels of RT (women were received a dose of 30 to 76 Gy), we compared the estimated maximum radiation dose to the contralateral breast with the estimated radiation dose at the site of the tumour in the contralateral breast between mutation carriers and non-carriers. The mean maximum RT dose and dose at the site of the tumour in the (contralateral) breast were comparable for those patients carrying a germline mutation and those without a germline mutation (Table 1). The dose distribution among carriers and non-carriers was also comparable, with an interquartile range for maximum RT dose of 1.0 to 1.8 Gy among carriers and 0.9 to 1.9 Gy among non-carriers, and an interquartile range for the dose at the site of the tumour of 1.3 to 2.2 Gy among carriers and 1.5 to 2.8 Gy among non-carriers.

The literature suggests that the increased risk of breast cancer due to radiation is not observed until at least 5 years after exposure [32,33]. When we subdivided all cases into two groups on the basis of the interval between first and second breast cancer (1 to 5 years and more than 5 years), we did indeed observe a difference between these groups (Table 5). The strongest effect was observed in women who developed a second primary breast tumour at least 5 years after radiotherapy; of these, 24% carried a germline mutation in one of the genes tested, in contrast with only 11% in those who did not receive any RT. Thus, at least 5 years after RT for the first breast cancer, women carrying a germline mutation in a DDRP gene had a significantly increased risk (OR 2.51, 95% CI 1.03 to 6.10; *p *= 0.049) of developing a radiation-associated CBC compared with women not carrying such a mutation.

## Discussion

This study is, to the best of our knowledge, the first to suggest that women with a germline mutation in a gene involved in DDRP have an increased risk of developing radiation-associated CBC, compared with women who are not carrying such a mutation. We examined the mutation frequency of *BRCA1*, *BRCA2*, *CHEK2 *and *ATM *in women who had developed a second primary breast tumour at least 1 year after a first breast cancer diagnosed before the age of 50 years, with or without RT for their first breast cancer. Such a case-only design is particularly well suited to the assessment of gene-environment interactions, although the independent effects of the genes involved or of radiation cannot be determined from these data [26,27]. ORs from such a design can be interpreted as the risk of developing radiation-associated disease for carriers compared with non-carriers. We present evidence for an interaction between mutations in the DDRP genes and radiation for the first breast cancer in the development of CBC.

Women with breast cancer have in general a threefold to fourfold increased risk of developing a new primary cancer in the contralateral breast [22]. The 15-year cumulative risk of developing CBC amounts to 10 to 13% [34-36]. *BRCA1 *and *BRCA2 *mutation carriers have an estimated CBC cumulative risk of 50 to 60% at the age of 70 years. The highest incidence is seen in the first 5 years after diagnosis of the primary breast cancer (12 to 33% per year), in contrast with 0.4 to 1% per year for breast cancer patients in general [37,38]. In a recent study, based on a consecutive series of breast cancer patients under 50 years of age, unselected for family history, we showed that CHEK2*1100delC carriers have a twofold increased risk of second breast cancer [39].

In the present study we found that mutation carriers have an additional excess risk, compared with non-carriers, of developing CBC during follow-up if they received RT for their first breast tumour. The increased risk due to RT exposure in carriers was mainly seen from 5 years after RT (Table 5). This is in accordance with the analysis of Boice and colleagues, who suggested previously that cancers resulting from an exposure to radiation would develop within predictable time windows; a latency period of at least 5 to 10 years was suggested for second primaries [32]. Metcalf and colleagues have shown that RT actually protects against local recurrences and ipsilateral breast cancer among *BRCA1 *and *BRCA2 *carriers [36].

Age at time of exposure is known to be a major determinant of the risk of breast cancer [40]. Our data also suggest that carriers in the younger age group at the time of exposure (less than 40 years old) showed the highest risk of developing radiation-associated CBC, although the difference from those above 40 years of age at RT was not significant (Table 4). Importantly, the mean and median ages at diagnosis of the first primary breast cancer were the same in the CBC patients with and without prior RT and in CBC patients with and without a germline mutation, resulting in the same median age at time of treatment in both groups (Table 1). Indeed, adjustment for age at first diagnosis did not affect our risk estimates.

The association between breast cancer risk and low-dose radiation has been a subject of debate. Recently we showed that diagnostic ionising radiation exposure from chest X-rays ma*y *be associated with a significantly increased breast cancer risk among women who carry *BRCA1 *or *BRCA2 *pathogenic germline mutations [4]. Comparable findings were reported for CHEK2*1100delC carriers by Bernstein and colleagues [21]. In the present study we included women exposed to higher doses from therapeutic RT, in whom the contralateral breast is exposed to about 1 to 10% of the total dose (namely 1 to 6 Gy), increasing the likelihood of detecting a gene-radiation interaction. The average (mean and median) maximum radiation dose (about 1.5 Gy) and the dose at the site of the tumour (about 1.8 Gy) measured in this study were found to be comparable between carriers and non-carriers. According to Preston and colleagues, the excess relative risk at 1 Gy is 2, implying that mutation carriers may have an excess relative risk of about 2.5 times this risk [16].

We increased the power of our analysis by investigating the effect of four breast cancer susceptibility genes together, considering them as a DNA repair gene group. These genes are all involved in DNA damage response triggered by damage induced by ionising radiation. An efficient response to DNA damage is essential for cellular life. The most detrimental form of damage is DNA double-strand breaks, which can be induced by ionising radiation and are lethal to the cell if not repaired. If repaired incorrectly, as a result of improper functioning of the repair machinery caused by mutations in the genes implicated, double-strand breaks can lead to carcinogenesis through translocation, inversions or deletions of genetic material. Although the relative impact of germline mutations in the separate genes studied in response to RT is unknown, all of these genes have been identified as crucial to the repair of RT-induced double-strand breaks. We therefore considered it justified to regard these genes together as a DNA repair gene group in our analyses. Although we screened for only a subset of all mutations in the four genes, there is no biological evidence that undetected mutations would have occurred more often in the non-RT group.

In a case-only study there must be independence between exposure (RT) and genotype (mutation) [26]. By selecting the RT-exposed and non-RT-exposed groups of CBC patients we might have consistently selected for those clinico-pathological factors determining the use of radiotherapy. If mutation carriers were more likely to receive RT for primary breast cancer, for example because of the presence of a family history of the disease, alerting the physician to a potentially more aggressive course of the disease, selection bias might arise in our case-only study. However, this was not the case, mutation carriers did not receive RT more often. Information about a family history of breast cancer was obtained by a mailed questionnaire and from medical records. In our study population 53% of the carriers reported breast cancer in the family (33% with an affected first-degree relative), in contrast with 40% in the non-carriers (29% with an affected first-degree relative). From the data collected from medical records and risk factor questionnaires of all women, we concluded that DDRP mutation carriers had clinico-pathological features (such as size and stage) similar to those of 'sporadic' cases. Adjustment for each of these factors in logistic regression analysis did not materially affect the risk estimates for mutation status. In addition, the percentages of patients receiving chemotherapy were comparable for mutation carriers and non-carriers. Consequently, the genetic factors (the genetic status was not known at time of treatment) under study did not affect treatment choice, justifying our assumption of independence between radiation and a mutation carrier status, which is needed for a case-only study.

In addition, in a related project studying the effect of *BRCA1*, *BRCA2 *and CHEK2*1100delC germline mutations on survival and disease outcome in a large cohort of unselected breast cancer patients (diagnosis before 50 years of age), we found that the tumour characteristics of CHEK2*1100delC carriers did not differ significantly from those of non-carriers. Importantly, in that same study we also found that the proportion of breast cancer patients receiving radiotherapy did not significantly differ between the CHEK2*1100delC carriers and non-carriers [39].

Although the effect of pathogenic DDRP gene mutations on breast cancer risk seems evident, the role of most *ATM *missense mutations remains unclear. Generally, the idea is that there are two groups of *ATM *heterozygotes in the general population, each with different cancer risks: those with a wild-type and a truncating mutation allele and those with a wild-type and a missense mutation allele. Several epidemiological studies have shown that relatives of patients with ataxia-telangiectasia heterozygotic for an *ATM *germline mutation have an increased risk of breast cancer [6]. Carriers of mutations predicted to encode a full-length ATM protein had cancer risks similar to those of people carrying truncating mutations, predicting that both type of mutation in these families are pathogenic for ataxia-telangiectasia as well as for breast cancer [6,41]. Most studies try to predict the relevance of a particular mutation on the basis of co-segregation in breast cancer families, the location in a functional domain or interference with the splicing machinery [42]. Nevertheless, only a few studies have presented the functional analyses necessary to assess the biological impact of unidentified variants found frequently in *ATM *[43]. We found that *ATM *missense mutations, those detected in our study, do not by themselves contribute to an increased risk of breast cancer after exposure to radiation. Although not significant, there might be an increased risk of developing CBC after RT when patients have both a pathogenic DDRP and an *ATM *missense mutation (Table 3). This trend suggests that *ATM *missense mutations might have a risk-modifying effect after radiation exposure.

It has been hypothesised that a multigenic model might explain breast cancer susceptibility in a large part of the population. It has been shown that, although mutations in certain genes had marginal or no associations with risk when studied in isolation, they showed significant association when combined with variant alleles in other genes [44]. It will be necessary to confirm the potential pathogenic-missense mutation (gene-gene) interaction findings of this study by a larger-scale study designed specifically to examine the joint effects of radiation exposure and genetic susceptibility of breast cancer risk (for example the Women's Environmental Cancer and radiation epidemiology ('WECARE') study [45]).

## Conclusion

Our data imply that there is a subgroup in the female population with increased susceptibility to radiation-induced breast cancer. The characterisation and identification of such a radiosensitive subgroup, if confirmed by others, will have implications for both diagnostic testing and the need for tailored treatment strategies. It provides a scientific basis for mutation analysis and subsequently, following the mutation analysis, an intensified follow-up protocol for mutation carriers. Identification of women susceptible to radiation damage will contribute to the risk/benefit assessment of radiation therapy versus alternative therapeutic options (such as breast-conserving surgery plus local radiotherapy or mastectomy).

## Abbreviations

CBC = contralateral breast cancer; CI = confidence interval; DDRP = DNA-damage repair pathway; OR = odds ratio; RT = radiation treatment.

## Competing interests

The authors declare that they have no competing interests.

## Authors' contributions

AB was involved in coordination of the study, performing the experiments and analysing results, and wrote the manuscript. LMB, AH and JU were involved in the mutation analysis experiments. AN was involved in patient selection, medical record and questionnaire abstraction and database management. FH participated in mutation identification management. MKS participated in the SPSS statistical and database analysis. JGMK participated in patient identification and selection. NSR participated in patient selection and provided radiation dosimetry. FEvL participated in the design and epidemiological analysis and LJV participated in the design and coordination of the study. All authors read and approved the final manuscript.

## Figures and Tables

**Table 1:** Tumour and patient characteristics, by DDRP mutation carrier status and previous RT exposure

Characteristic	Mutation positive (*n *= 51)		Mutation negative (*n *= 196)	
	Previous RT	No previous RT	Previous RT	No previous RT
Age at first BC, years	40.3	42.4	41.4	42.3
Age at second BC, years	48.1	51.8	51.1	54.2
Chemotherapy, *n *(percentage)	11 (27)	1 (10)	28 (22)	5 (7)
Tumour size, cm	1.8	2.9	2.2	2.3
Lymph node positive, *n *(percentage)	12 (29)	1 (10)	41 (32)	4 (6)
RT dose^a^, Gy				
Mean (range)	1.49 (0.27–3.73)		1.55 (0.24–5.61)	
Median	1.32		1.40	
Third quartile	1.79		1.89	

DDRP, DNA-damage repair pathway; BC, breast cancer; RT, radiation treatment. ^a^Radiation dose to the site of the breast where the tumour had developed.

**Table 2:** Frequencies of contralateral breast cancer patients CBC carrying apathogenic DNA-damage repair pathway mutation

Parameter	Breast cancer < 50 years^a ^(percentage)	All CBC patients (*n *= 247)		Previous RT (*n *= 169)		No RT (*n *= 78)		Carriers versus non-carriers		
		*n*	Percentage	*n*	Percentage	*n*	Percentage	OR for CBC after RT	95% CI	*p*
DDRP mutated	~10	51	21	41	24.3	10	12.8	2.18	1.03–4.62	0.043
*CHEK2*	1–2	15	6.1	13	7.7	2	2.6	3.17	0.69–14.39	
*BRCA1*	~4–5	27	11.3	20	11.8	7	9	1.36	0.55–3.37	
*BRCA2*	~0.5–1	5	2	4	2.4	1	1.3	1.87	0.21–16.98	
*ATM *truncating	~1–2	4	1.6	4	2.4	0	0	-	-	
						1^b^	1.3	1.87	0.21–16.98	

Results for all contralateral breast cancer (CBC) patients, and patients stratified by previous radiation exposure, are presented. Odds ratio (OR) and 95% confidence interval (CI) are given for mutation carriers versus non-carriers to develop radiation-associated breast cancer. DDRP, DNA-damage repair pathway; RT, radiation treatment. ^a^Published results; ^b^Hypothetical assumption of one case.

**Table 3:** Frequencies of CBC patients carrying pathogenic DNA-damage repair pathway and *ATM *missense mutations

Parameter	All CBC patients (*n *= 247)		Previous RT (*n *= 169)		No RT (*n *= 78)		Carriers versus non-carriers		
	*n*	Percentage	*n*	Percentage	*n*	Percentage	OR for CBC after RT	95% CI	*p*
DDRP mutated	51	21	41	24.3	10	12.8	2.18	1.03–4.62	0.043
All patients with only a pathogenic DDRP mutation	37	15	29	17.2	8	10.3	1.8	0.79–4.17	
Patients with both a pathogenic DDRP and an *ATM *missense mutation	14	5.7	12	7.1	2	2.6	2.90	0.63–13.30	
Patients with only an *ATM *missense mutation	29	11.7	21	12.4	8	10.3	1.24	0.52–2.94	

Results for all contralateral breast cancer (CBC) patients, and patients stratified by previous radiation exposure, are presented. Odds ratio (OR) and 95% confidence interval (CI) are given for mutation carriers versus non-carriers to develop radiation-associated breast cancer. DDRP, DNA-damage repair pathway; RT, radiation treatment.

**Table 4:** Frequencies of CBC patients carrying apathogenic DNA-damage repair pathway mutation

Age (years)	All CBC patients		Previous RT		No RT		Carriers versus non-carriers	
	*n*	Percentage	*n*	Percentage	*n*	Percentage	OR for CBC after RT	95% CI
<40 (*n *= 91)	22/91	24	19/67	28	3/24	12	2.77	0.74–10.39
40–50 (*n *= 156)	29/156	18.6	22/102	21.6	7/54	12.9	1.85	0.73–4.65

Results for all contralateral breast cancer (CBC) patients, and patients stratified by previous radiation exposure, are presented, according to age at diagnosis of first primary breast cancer (before the age of 40 years or between 40 and 50 years of age). Odds ratio (OR) and 95% confidence interval (CI) are given for mutation carriers versus non-carriers to develop radiation-associated breast cancer. RT, radiation treatment.

**Table 5:** Frequencies of CBC patients carrying apathogenic DNA-damage repair pathway mutation

Interval (years)	All CBC patients		Previous RT		No RT		Carriers versus non-carriers		
	*n*	Percentage	*n*	Percentage	*n*	Percentage	OR for CBC after RT	95% CI	*p*
1–5 (*n *= 66)	15/66	22.7	12/49	24.5	3/16	18.8	1.41	0.34–5.78	
>5 (*n *= 181)	36/181	19.9	29/120	24.1	7/62	11.3	2.51	1.03–6.10	0.049

All contralateral breast cancer (CBC) patients, and patients stratified by previous radiation exposure are presented, according to interval between first and second primary breast cancer (less than 5 and more than 5 years). Odds ratio (OR) and 95% confidence interval (CI) are given for mutation carriers versus non-carriers to develop radiation-associated breast cancer. RT, radiation treatment.
